# Diagnostic Value of Cone-Beam Computed Tomography and Periapical Radiography In Detection of Vertical Root Fracture

**Published:** 2015-03-18

**Authors:** Fatemeh Ezzodini Ardakani, Seyed Hossein Razavi, Mehdi Tabrizizadeh

**Affiliations:** a *Department of Oral and Maxillofacial Radiology, Dental Faculty, Shahid Sadoughi University of Medical Sciences, Yazd, Iran; *; b * Department of Endodontics, Dental Faculty, Shahid Sadoughi University of Medical Sciences, Yazd, Iran*

**Keywords:** Cone-Beam Computed Tomography, CBCT, Periapical Radiography, Vertical Root Fracture

## Abstract

I**ntroduction: **Vertical root fracture (VRF) is the longitudinal fracture of the root in endodontically treated teeth. Considering the limitations of two-dimensional radiographic images in detection of VRF and introduction of cone-beam computed tomography (CBCT), this study was designed to find the sensitivity, specificity and accuracy of CBCT and periapical (PA) radiography in detection of VRFs. **Methods and Materials: **This was a cross-sectional *in vitro* study on 80 extracted human single canal teeth including 40 maxillary and 40 mandibular teeth. After standardized endodontic treatment of the roots, VRF was induced in half of the teeth in each group, and other half were left without fracture. Teeth were inserted in dry maxillary and mandibular alveoli. PA radiographs and CBCT images were taken from the specimens. Data were analyzed with SPSS software. The McNemar test was used to evaluate the sensitivity, specificity and accuracy of images, and kappa coefficient was used to assess the degree of agreement between the observers. The level of significance was set at 0.05. **Results: **Sensitivity and specificity values of CBCT were 97.5% and 95%, respectively. However, for PA radiography the sensitivity and specificity were 67.5% and 92.5%, in order of appearance. Accuracy of CBCT (96.25%) and PA radiography (80%) in both jaws were significantly different (*P*=0.022). Two methods were not significantly different when testing specificity (*P*=0.298).** Conclusion: **This study showed that the sensitivity and accuracy of CBCT in detection of vertical root fracture are higher than periapical radiography. CBCT can be recommended to be used in detection of vertical root fractures.

## Introduction

Vertical root fracture (VRF) is the longitudinal fracture of the root in endodontically treated teeth [1]. It usually begins from the apex and extends towards the coronal segment of the root. The fracture originates from the internal wall of the canal and extends outward. VRF usually do not present with specific signs, and it can be certainly detected by the observation of fracture line [2-4]. VRF can be observed in 3.69 to 20% of the teeth with history of root canal therapy [5-7] and is most frequently seen in second maxillary premolars (27.2%), and mesial roots of mandibular molars (24%) [8]. These fractures are formed mostly in buccolingual direction [9]. Two major causes of VRF are post placement and extra ordinary condensation forces during obturation of the canal.

Two-dimensional (2D) radiography has some limitations in detection of VRF, as the fracture line can be detected only when it is located in the path of the radiation; therefore three-dimensional radiographic techniques are probably more successful in this regard [10, 11]. Considering the importance of detecting VRF in the maintenance prognosis of teeth and difficulty in clinical and radiologic diagnosis of this complication, finding a method with higher accuracy for detection of VRF is important. 

In late 1990s, Cone-beam computed tomography (CBCT) was introduced and it was free of the limitations and obstacles of previous computer-aided methods including CT scan [12]. In dentistry this technique has got some advantages over CT scan such as lower price, lower exposure duration, higher resolution, applicability and stronger software for reconstruction of images. The three-dimensional (3D) nature of CBCT can provide more information about dental structure compared to conventional radiography [12]. CBCT has been introduced as a method for detection of VRF.

**Figure 1:**
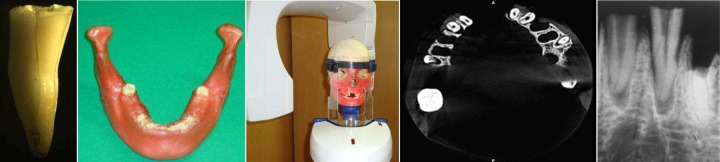
*A)* A tooth with vertical root fracture under stereomicroscope (×10 magnnification); *B)* Jaws used in the study were covered by two layers of a boxing wax for reconstruction of soft tissue; *C)* One of the mandibles and maxillae used in CBCT device; *D)* Axial plane of CBCT showing two teeth with vertical root fracture; *E)* A tooth with vertical root fracture in a periapical radiograph

VRFs lack specific hallmarks in conventional radiography, so its detection is difficult. Studies have been performed to assess the accuracy of different radiologic methods such as 2D digital and film-based radiography and 3D methods such as conventional CT and CBCT. 

In a cross-sectional study on 80 molars and premolars, Hassan *et al. *[13] compared the accuracy of periapical (PA) radiography and CBCT in detection of VRF. They also assessed the effect of canal restoration in detection of the fracture. They found a higher accuracy of CBCT in comparison with radiography for detection of VRF. On the other hand, Edlund *et al.* [14] found that CBCT is 88% sensitive and 75% specific for detection of VRF. They assessed VRF in patients with clinical signs suggestive of VRF. 

This *in vitro* study aimed to compare the diagnostic value (sensitivity, specificity and accuracy) of CBCT and PA radiography in detection of VRF. 

## Materials and Methods

This cross-sectional *in vitro* study was conducted on 80 single-canal human teeth (40 maxillary and 40 mandibular teeth) that were extracted due to decay, orthodontic treatment plan or periodontal diseases.

After disinfection with 5.25% sodium hypochlorite (NaOCl) the crowns were cut at the level of cemento-enamel junction (CEJ). Then all root canals were instrumented with a circumferential filing technique using hand K-files (Dentsply

**Table 1:** Degree of agreement among three observers during assessment of VRF using CBCT and periapical (PA) radiography

**Pairs of observers**	**Imaging method **	
	**CBCT**	**PA**
**First and second**	0.68	0.59
**Second and third**	0.82	0.62
**First and third**	0.71	0.59

Maillefer, Ballaigues, Switzerland) and step-back preparation of the canals. After filing and flaring, 40 teeth were included in the study as the control group and 40 (20 maxillary and 20 mandibular) teeth were randomly selected for inducing VRF (*n*=40). The roots were covered by a thin layer of red wax. For all teeth, two third of the root length was mounted in acrylic blocks from apical region. The method of inducing VRF was similar to that used by Hassan *et al.* [15]. In this method, a sharp chisel was placed perpendicular to the root canal and fracture was created by fine impacts of a hammer. If the two parts of tooth were splited due to the fracture, the specimen was excluded. Direct observation under a stereomicroscope (Wild M5A, Heerbrugg, Switzerland) with 10× magnification was used as the gold standard for detection of the fracture (Figure 1A). The other specimens in the control group were also assessed by similar method to assure that they were fracture free. 

The teeth were randomly numbered before imaging by a person who was blind to observation and interpretation of PA and CBCT images. Then the specimens were placed in two dry mandibular and maxillary alveoli containing dental sockets. For soft tissue reconstruction, the bony surfaces were covered by two layers of boxing wax (Figure 1B and C) before taking PA and CBCT images (Figure 1E and D). 

**Table 2:** The rate of VRF detection by CBCT in comparison with the gold standard (observations under a stereomicroscope [VRF^-^=without fracture (socres 1 and 2) and VRF^+^=with fracture (socres 3 and 4)

**Gold standard**	**CBCT**		**Total**
	**VRF** ^+^	**VRF** ^-^	
**VRF** ^+^	39	2	41
**VRF** ^-^	1	38	39
**Total **	40	40	80

**Table 3:** The rate of VRF detection by CBCT in comparison with periapical (PA) radiography [VRF^-^=without fracture (socres 1 and 2) and VRF^+^=with fracture (socres 3 and 4)

**Gold standard**	**CBCT**		**Total**
	**VRF** ^+^	**VRF** ^-^	
**VRF** ^+^	27	3	30
**VRF** ^-^	13	37	50
**Total **	40	40	80

**Figure 2:**
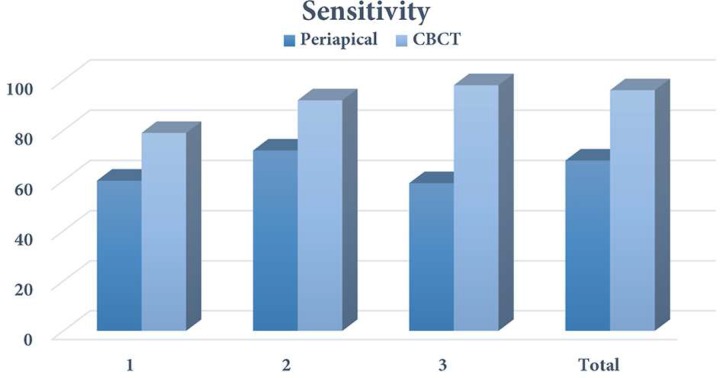
Sensitivity of CBCT and periapical radiography in detection of vertical root fracture between different observers (1, 2 and 3)

CBCT images were taken by Planmeca Promax 3D (Planmeca, Helsinki, Finland). The exposure settings were 66 kVp, 8 mA and 12 sec. Field of view (FOV) of the device was 8×8 cm. Volume reconstruction was done by Romexis Viewer (Planmeca, Helsinki, Finland). 

PA images were taken by Planmeca EC Proline (Planmeca, Helsinki, Finland) with exposure settings of 60 kVp and 8 mA on size 2 E-speed Kodak films (Eastman-Kodak Co., Rochester, NY, USA). All images were developed by an automatic film processing device (Velox, England) for 4 min. 

The obtained images (including radiographies and CBCTs) were interpreted by three observers (an oromaxillofacial radiologist, an endodontist and an oromaxillofacial radiology resident). None of the observers were aware of the real results of the images. CBCT images were assessed in three planes (axial, coronal and sagittal). The following 5-point Likert scale was used for interpretation of the images: *score 1*-certainly without VRF; *score 2*-probably without VRF; *score 3*-certainly with VRF; *score 4*-probably with VRF and *score 5*-uncertain presence of VRF.

The results were recorded in a checklist according to this scale. The data were analyzed using the SPSS software (SPSS version 19.0, SPSS, Chicago, IL, USA). For identification of sensitivity and specificity, the results of each method were compared with the results of gold standard (observations under a stereomicroscope with 10× magnification) and McNemar test was used for analysis.

The kappa coefficient was calculated to find the amount of agreement between the methods. Inter-rater reliability was used to examine the agreement between two observers on a categorical variable. The kappa coefficient <0.4, 0.4-0.75 and >0.75 were considered as weak, intermediate, and strong agreement, respectively.

**Figure 3:**
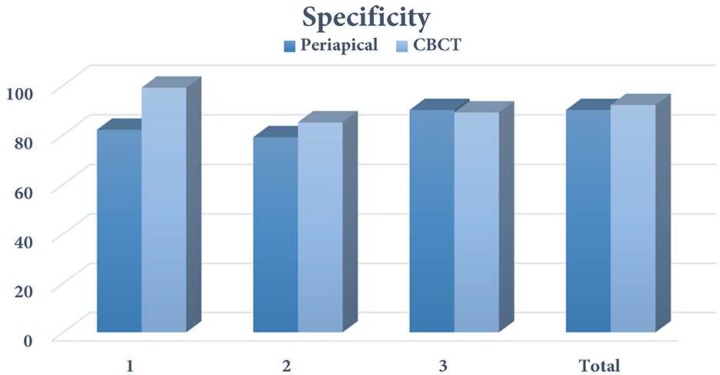
Specificity of CBCT and periapical radiography in detection of vertical root fracture between different observer (1, 2 and 3)

## Results

Table 1 shows the degree of agreement among three observers for CBCT and PA radiography in assessment of VRF. Table 2 shows the results of VRF detection by CBCT in comparison with gold standard. Considering the defined categories for Likert scale, in this study scores 1 and 2 were considered as "without fracture" and scores 3 and 4 as "with fracture"; and no score 5 was observed. Diagnostic indices were measured according to 2D tables of the results of gold standard (direct vision with microscope) and the results of CBCT and PA (divided into with and without fracture). Sensitivity of CBCT for detection of VRF was 95% and 100% in mandible and maxilla, respectively. The specificity of this method for detection of VRF in maxilla and mandible was 95%, and its accuracy was 95% in mandible and 97.5% in maxilla. Overall sensitivity, specificity and accuracy of CBCT for detection of VRF were 97.5%, 95%, and 96.25%, respectively.

Table 3 shows the results of VRF detection by PA radiography in comparison to gold standard. Sensitivity of PA radiography for detection of VRF was 70% and 65% in mandible and maxilla, respectively. The specificity of this method for detection of VRF was 90% and 95% in mandible and maxilla, respectively and its accuracy was 80% in mandible and 80% in maxilla. Overall sensitivity, specificity and accuracy of PA radiography for detection of VRF were 67.5%, 92.5%, and 80%, respectively.

Table 4 compares the sensitivity and specificity of CBCT and PA radiography in detection of VRF. *P*-values have been measured by kappa test (two dependent rates). 

**Table 4:** Comparison of the sensitivity (Sen), specificity (Spe) and accuracy (Acc) of CBCT and periapical (PA) radiography in detection of VRF in mandible(Mand) and maxilla (Max)

**Imaging method**	**Sen**	**Spe**	**Acc in Mand**	***P-value***	**Acc in Max**	***P-value***	**Overall Acc**	***P-value***
**CBCT**	97.5	95	95	0.028	97.5	0.021	96.25	0.021
**PA**	97.5	95	80		80		80	

## Discussion

In this *in vitro *study, the accuracy of two imaging methods (CBCT and PA radiography) in detection of VRF was compared. The results showed that the overall sensitivity of CBCT was significantly higher than PA radiography, but their specificity was not significantly different. We chose film-based radiography instead of digital images, because several studies showed that there is no significant difference between digital and film-based radiography in detection of VRF [16-20].

In 2D radiography for detection of VRF, the direction of X-ray should be parallel (±4^°^) to the fracture line [21]. Radiographic features of VRF include: a visible fracture line in radiography, separation of root fragments, a space besides root filling and canal wall, vertical bone loss [22] and a characteristic diffused or halo/J type radiolucency surrounding the root [23].

Studies showed that VRF can be observed in up to 20% of extracted teeth [17-19]. Therefore early detection of VRF is critical to prevent resorption of surrounding tissue and bone. In this study, CBCT and intra-oral PA radiography with E-speed film were compared regarding their accuracy in detection of VRF. The current study showed that diagnostic accuracy of CBCT for detection of VRF is higher than PA radiography. 

Before current popularity of CBCT in dentistry, some studies assessed other 3D radiographic techniques such as conventional CT scan in detection of VRF. Youssefzadeh *et al.* [24] assessed the detection rate of VRF by conventional radiography and CT scan. They reported the accuracy of CT scan for detection of VRF to be higher than conventional PA radiography; but considering high irradiation dose, it seems that this method is not appropriate for detection of VRF. 

The results of current study are consistent with the results of the study conducted by Valiozadeh *et al.* [20] who examined the accuracy of conventional and digital radiography, and CBCT in detection of VRF in single-canal teeth. They found that VRF is more accurately detected by CBCT than conventional and digital radiography. However the specificity of conventional radiography for detection of VRF in the present study was higher. 

The results of the current study are in agreement with the results of a clinical trial on 29 patients by Edlund *et al. *[14]. They found that sensitivity and specificity of CBCT in detection of VRF was 88% and 75%, respectively. 

In the current study there were no filling substances (such as gutta-percha) in the root canal, which may increase the diagnostic strength of the radiographic method in comparison to clinical studies. Besides, presence of post or metallic substances can increase the probability of metallic artifact in CBCT images [25]. 

The results of the present study were consistent with the results reported by Bernardes *et al.* [26] on 20 patients with possible VRF detected with either CBCT or PA radiography. In separate studies, Varshosaz *et al.* [27] and Hassan *et al.* [13] compared the accuracy of CBCT and digital radiography in detection of VRF and reported a higher accuracy for CBCT.

Routine radiographic methods has some disadvantages in detection of VRF: the angle of X-ray in relation to fracture line, possible superimposition of the structures, and lack of the third dimension. In CBCT images it is possible to observe the images in all three dimensions and evaluate the fracture line in different planes (coronal, axial, and sagittal) with a high contrast [13]. The results of the current study also confirm the fact that VRF can be more accurately detected with CBCT techniques. However, the high cost remains as the main disadvantage of CBCT imaging.

## Conclusion

Generally, the test with higher sensitivity is chosen to detect a lesion. However, due to the high cost and radiation dose of CBCT and considering the high specificity of periapical radiography (few false positive cases), if periapical radiography shows VRF there is no need for CBCT confirmation. Nevertheless when periapical radiography fails to detect a suspected VRF, CBCT is recommended as the next diagnostic method.
